# Detoxification Combining Fasting with Fluid Therapy for Refractory Cases of Severe Atopic Dermatitis

**DOI:** 10.1155/2013/561290

**Published:** 2013-08-06

**Authors:** Kyu Seok Kim, Hae Jeong Nam

**Affiliations:** Department of Ophthalmology, Otorhinolaryngology and Dermatology of Korean Medicine, College of Korean Medicine, Kyung Hee University, 1 Hoegi-dong, Dongdaemun-gu, Seoul 130-701, Republic of Korea

## Abstract

To introduce and determine the clinical benefits of a detoxification program that combines fasting with fluid therapy for refractory cases of severe atopic dermatitis (AD), we performed a retrospective chart review of inpatients with AD from March 2010 to February 2012 at the Department of Ophthalmology, Otorhinolaryngology and Dermatology of Korean Medicine in the Kyung Hee Medical Center. Patients were treated with the detoxification program, which combined fasting with fluid therapy, and herbal medicine, herbal wet wrap dressings, or acupuncture treatment when clinically necessary. The primary outcome was the SCORAD total index. The secondary outcome was the pruritus visual analogue scale (VAS) score in SCORAD as evaluated by a trained dermatology specialist. Among the 130 inpatients that have done detoxification, 7 patients met the inclusion criteria. The mean total SCORAD scores significantly decreased from 64.67 ± 11.72 to 26.26 ± 11.01 (*P* = 0.018) after the detoxification program. There was also a significant decrease in VAS score for pruritus from 8.00 ± 1.16 to 2.57 ± 0.98 (*P* = 0.016) between admission and discharge. We suggest that fasting with fluid therapy as a complementary and alternative treatment method may provide some benefits for patients with refractory cases of severe atopic dermatitis.

## 1. Introduction

Atopic dermatitis (AD) is a chronic relapsing inflammatory skin condition with extensive pruritus, erythema, excoriations, and scaly skin lesions [1]. Westernized medicine has conventionally used a combination of emollients, corticosteroids, antibiotics, calcineurin inhibitors, UV phototherapy, and systemic immunomodulating therapies like cyclosporine and interferon gamma-1b [1, 2]. Long-term conventional treatments for AD are occasionally difficult because of the chronic, recurrent nature of AD; thus, there is an increasing need to find better therapies that have minimal side effects [2, 3]. Lately, traditional Chinese medicine (TCM) with natural herbs and acupuncture has been regarded as a new therapy for AD that could minimize the use of corticosteroids and their side effects. However, TCM also has some problems, as some patients with severe AD who are refractory to conventional therapy developed significant side effects to TCM. 

Detoxification, a part of complementary and alternative medicine, is described as working by releasing “toxins” from the body contaminated by metabolites and environmental toxins or by overindulgence and an insalubrious lifestyle [4]. Among the various methods for detoxification, fasting has an inhibitory effect on allergic dermatitis in experimental mouse models [5–7]. Furthermore, a clinical study has suggested a positive relationship between weight loss through repeated short-term fasting and symptom improvement in patients with AD [8]. Based on these previous studies, we applied the detoxification program, which combined fasting with fluid therapy, to refractory cases of severe AD. 

The aim of this study was to introduce and evaluate the clinical benefits of this detoxification program on AD by performing a retrospective chart review of inpatients with severe AD.

## 2. Methods 

### 2.1. Patients

We conducted a retrospective chart review of inpatients who have done detoxification program from March 2010 to February 2012 at the Department of Ophthalmology, Otorhinolaryngology and Dermatology of Korean Medicine in the Kyung Hee Medical Center in Seoul and then selected refractory cases of severe AD. Eligibility criteria for inclusion were (1) a diagnosis of AD according to the UK Working Party's Diagnostic Criteria [9], (2) an age of 18 to 40 years, (3) admission required because of refractory pruritus or lifestyles that could exacerbate symptoms, and (4) a Scoring AD (SCORAD) index greater than 50. Verbal informed consent was obtained from each patient. Data was stored to safeguard confidentiality in password protected computer. Only one investigator had access to harvested patient data and when no longer needed data would be destroyed.

This study was approved by the Institutional Review Board of Kyung Hee Oriental Medical Center (IRB approval number KOMCIRB 2013-02).

### 2.2. Outcome Measurement

Primary outcome was the SCORAD total index at each admission and discharge day. Secondary outcomes were the pruritus self-assessment score changes of AD-related itching/scratching at each admission and discharge day, adverse changes in daily vital signs (BP, pulse, and temperature), and peripheral blood glucose level.

### 2.3. Adverse Events and Safety Monitoring

All unpredictable adverse events related to the detoxification program were reported to two Korean medical doctors (KMD and one medical doctor (MD) in the Department of East and West Integrated Medicine. Safety was assessed by the reporting of clinical laboratory tests, vital signs, and adverse events. Clinical laboratory tests, including AST/ALT, BUN/creatinine, red blood cell (RBC) count, white blood cell (WBC) count, hemoglobin, hematocrit, mean cell volume (MCV), mean cell hemoglobin (MCH), mean cell hemoglobin concentration (MCHC), number of platelets, and number of differentiated cells, were determined at each admission and discharge day. Vital signs of inpatients were checked with monitoring of adverse events (nausea/vomiting, fatigue, allergic reaction, and any adverse events related to the detoxification program) three times a day.

### 2.4. Intervention

#### 2.4.1. Fasting

**Figure 1:**
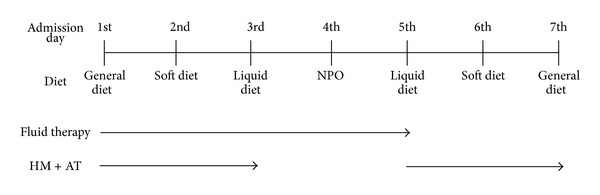
Flow chart of hospitalization program combining very low-calorie diet and fluid therapy. Abbreviation: HM, herbal medicine (decoction, ointment, or wet wrap dressing type); AT, acupuncture treatment; NPO, nothing per os.

The detoxification program consists of three stages: (1) very low-calorie diet, (2) fasting, and (3) convalescence (Figure 1). On admission to the hospital, we confirmed that the detoxification program was appropriate for the patients with atopic dermatitis through examinations like blood tests or electrocardiograms. Before the fasting stage, patients were prescribed a very low-calorie diet for three meals. At that time, we recommended that patients take a vermicide to empty the intestines. If a patient did not have a bowel movement before the fasting stage, we administered an enema or prescribed herbal medicine to help with bowel movements. After a very low-calorie diet for three meals, no food was given to inpatients for three meals during the fasting stage. After the fasting stage, we again prescribed a very low-calorie diet for three meals. Two days before hospital discharge, we prescribed a general diet in order to examine the skin condition of patients to assess for signs like itching or scaling after being exposed to a general diet.

#### 2.4.2. Fluid Therapy

From the day of admission to two days before discharge, we supplied about one liter of fluid per day (normal saline solution) to inpatients. If a patient complained of severely dry skin, it was possible to increase the amount of fluid. We examined the patients when fluid therapy was stopped.

#### 2.4.3. Herbal Medicine and Acupuncture Treatment

We prescribed herbal medicine (types of decoction, ointment, or wet wrap dressing) or acupuncture treatment if necessary. Usually, acupuncture treatment was provided twice per day at acupuncture points both sides of LI4, LI11, SP10, SP6, and LR3 to control the skin inflammation of AD patients. The herbal medicine was a decoction of plant material, including *Rehmannia glutinosa, Angelica gigas, Paeonia japonica, Cnidium officinale* Makino, *Sophora flavescens* Solander ex Aiton, and *Spirodela polyrhiza.* We decocted 0 to 12 grams of each plant material with purified water as a daily dose according to patient progress. This daily dose was administered three times a day after each meal. We also used a decoction of plant material for the herbal wet dressings, including *Aloe vera*. Four or five layers of sterilized gauze were hydrated sufficiently with the decoction and were applied immediately to the AD lesions for 15 minutes. Herbal wet dressings were applied once or twice per day according to symptom severity. We applied the herbal medicine for AD only during the very low-calorie diet stage, not during the fasting stage. If some patients were sensitive, we did not apply acupuncture treatment to them during the fasting stage. 

### 2.5. Statistical Analysis

Analyses were performed using SPSS version 17.0 for Windows. Data are presented as mean ± standard deviation (SD). The statistical calculation for Wilcoxon signed-rank test, a nonparametric method, was performed for the mean change of the SCORAD total index and the pruritus self-assessment score changes of AD-related itching/scratching between admission and discharge because assumptions of normality were violated. All differences were considered significant at *P* < 0.05.

## 3. Results 

### 3.1. Patients

**Figure 2:**
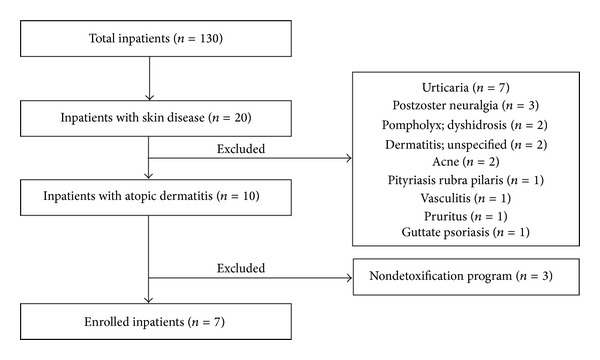
Flow chart of subjects' inclusion process.

Among the 130 inpatients that have done detoxification, a total of 7 patients were included in this study (Figure 2). Clinical characteristics of patients on admission day are summarized in Table 1. The mean age of the 7 inpatients was 24 years (range 19–30). Hospitalization lasted an average of 9.29 days (range 7–15). Serum immunoglobulin E level was an average of 4934 IU/mL (range 18–14000). Eosinophil count was an average of 854.29 cells/mcl (range 130–1700). Antistreptolysin O titer level was an average of 183.29 IU/mL (range 101–284).

### 3.2. Primary and Secondary Outcomes

**Figure 3:**
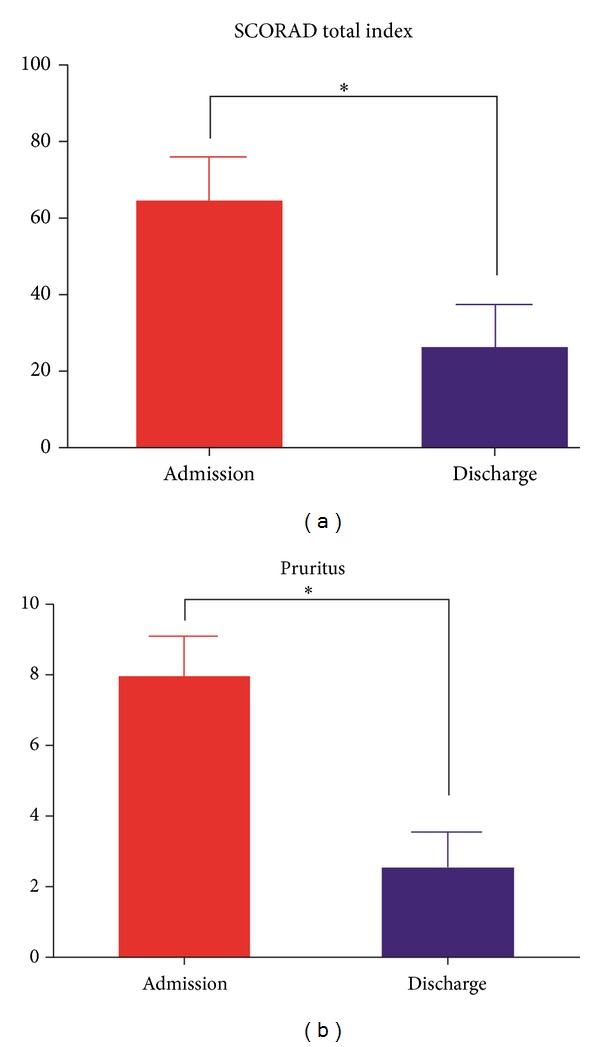
The mean change of SCORAD index and VAS score for pruritus after the detoxification program.

The primary outcome, which was the SCORAD total index at each admission and discharge day, significantly decreased from 64.67 ± 11.72 (extent: 45.14 ± 29.32/intensity: 11.86 ± 1.95) to 26.26 ± 11.01 (extent: 34.14 ± 25.77/intensity: 5.57 ± 2.07) (*P* = 0.018). There was also a significant decrease in a secondary outcome, the pruritus self-assessment score changes of AD-related itching/scratching, from 8.00 ± 1.16 to 2.57 ± 0.98 (*P* = 0.016) between admission and discharge day (Figure 3).

### 3.3. Safety Evaluation

No adverse events were reported. Mean levels in systolic and diastolic blood pressure, respiratory rate, heart rate, and body temperature were similar at all measurement times. Also, clinical laboratory tests were similar between admission and discharge day.

## 4. Discussion

AD, especially when unmanageable, can be distressing and can reduce quality of life. Quick and effective treatments are necessary. Unfortunately, conventional treatments, whether western medicine or TCM, often fail to relieve symptoms of AD [10]. Therefore, we modified previous studies of fasting and developed the detoxification program, which combined fasting and fluid therapy, for inpatients with refractory cases of severe AD. 

Fasting for a certain period of time can eliminate body waste, diminish gastrointestinal irritation, and refresh digestive and respiratory organs. In particular, inhibition of gastrointestinal irritation helps repair the mucous membrane and blocks any supply of unwholesome food, which can be allergens, for a certain period of time [11]. However, during the fasting stage, moisture and electrolytes can be lost. Accordingly, fluid therapy is needed to replenish the body with moisture and electrolytes [12]. A 0.9% hypertonic solution has some benefits because it is distributed first to the extracellular space. Although there is no scientific evidence on the pathophysiology, a large amount of fluid supply promoted recovery of postoperative or burn patients [13]. 

To apply detoxification to patients, careful attention is required in the following cases: (1) patients with gastric acid control disorders, (2) patients with hypoglycemia accompanied by vertigo or cold sweats, (3) patients with constipation, and (4) patients with anemia. 

The typical period of detoxification is usually 7 days. If necessary, the period can be longer or shorter than 7 days, but the fasting state should not be longer than 3 consecutive days. Sidedishes have to be supplied to patients 1 or 2 days after a low-calorie diet not immediately after finishing fasting. Also, to calculate the fluid supply for patients, urinary and skin loss of water must be considered [14]. Although there were no cases in this study, based on experience in clinical practice, the symptoms of patients with AD, such as night-time itching, can be aggravated during the third very low-calorie meal or second fasting meal. However, these symptoms improve after about two to three days. Therefore, we should explain to patients that symptoms related to AD can deteriorate during the period of about seven days after the fasting diet. We also have to explain to patients that symptoms related to AD can worsen if unwholesome foods, which can be allergens, are introduced after discharge from the hospital. 

In experimental animal studies, fasting protected or diminished the distress level of autoimmune disease and allergy [5–7]. In clinical studies, fasting [15] and a low-calorie diet [16] have been reported to be effective in patients with rheumatoid arthritis. A review study found that one randomized trial concluded that weight loss may have some benefits for relieving asthma in overweight and obese patients [17]. Other studies indicated that fasting and DR can protect the increase of allergen-specific IL-4-producing T cells and suppress the allergic reaction [5] and in humans can ameliorate T-cell function [18].

A low-energy diet reduced inflammatory symptoms and oxidative damage in patients with AD [19]. A positive relationship between weight loss through short-term fasting and symptom improvement in patients with AD has also been reported [8]. These results are consistent with our findings after the detoxification program, which combined fasting with fluid therapy, for inpatients with AD. 

However, there are still few data about fasting and fluid therapy in patients with AD. Numerous well-designed clinical studies, such as randomized controlled studies, are needed to acquire definite evidence on the effects of detoxification programs for inpatients with refractory cases of severe AD.

## 5. Conclusions

In this study, fasting with fluid therapy was effective for inpatients with refractory AD compared to conventional therapeutic modalities. The detoxification program, which combined fasting with fluid therapy, may be used as a novel treatment in refractory cases of severe AD. 

## Figures and Tables

**Table 1:** Patient summary.

Case	Age	Gender	Other atopy	Duration (unit: days)			Western medicine	HM type	AT	Serum IgE	Eosin count	ASO titer
				Hospitalization	VLCD Program	Fluid therapy						
1	19	Male	AR, AC, and food allergy (crab)	7	7	4	None	Decoction + ointment	Yes	1680	240	116
2	30	Male	AR, AC, and asthma	7	7	4	None	Decoction + ointment	Yes	14000	1340	273
3	20	Female	AR	12	8	5	Topical steroid	Decoction + ointment + wet wrap dressing	Yes	475	1510	284
4	22	Male	AR, food allergy (soybean)	8	8	4	None	Decoction + ointment	Yes	460	None	106
5	29	Male	ND	15	12	5	None	Decoction + ointment + wet wrap dressing	Yes	<18	130	202
6	25	Male	Food allergy	8	8	4	No response to topical and systemic steroid therapy	Decoction + ointment + wet wrap dressing	Yes	391	1700	101
7	23	Female	SD	8	8	4	None	Decoction + ointment	Yes	2400	210	201

*AR: allergic rhinitis; AC: allergic conjunctivitis; ND: nummular dermatitis; SD: seborrheic dermatitis; VLCD: very low calorie diet; HM: herbal medicine; AT: acupuncture treatment; IgE: immunoglobulin E; ASO: antistreptolysin O.

## References

[1] Berke R, Singh A, Guralnick M (2012). Atopic dermatitis: an overview. *The American Family Physician*.

[2] Dinicola C, Kekevian A, Chang C (2013). Integrative medicine as adjunct therapy in the treatment of atopic dermatitis-the role of traditional Chinese Mmedicine, dietary supplements, and other modalities. *Clinical Reviews in Allergy and Immunology*.

[3] Mizawa M, Makino T, Hikiami H, Shimada Y, Shimizu T (2012). Effectiveness of keishibukuryogan on chronic-stage lichenification associated with atopic dermatitis. *ISRN Dermatology*.

[4] Ernst E (2012). Alternative detox. *British Medical Bulletin*.

[5] Yamazaki K-I, Kato-Nagaoka N, Suzuki T, Shida K, Nanno M (2009). Immune deviation and alleviation of allergic reactions in mice subjected to dietary restriction. *Bioscience, Biotechnology and Biochemistry*.

[6] Kouda K, Iki M (2010). Beneficial effects of mild stress (Hormetic Effects): dietary restriction and health. *Journal of Physiological Anthropology*.

[7] Nakamura H, Kouda K, Tokunaga R, Takeuchi H (2004). Suppressive effects on delayed type hypersensitivity by fasting and dietary restriction in ICR mice. *Toxicology Letters*.

[8] Nakamura H, Shimoji K, Kouda K, Tokunaga R, Takeuchi H (2003). An adult with atopic dermatitis and repeated short-term fasting. *Journal of Physiological Anthropology and Applied Human Science*.

[9] Williams HC, Burney PGJ, Pembroke AC, Hay RJ (1994). The U.K. Working Party’s diagnostic criteria for atopic dermatitis. III. Independent hospital validation. *British Journal of Dermatology*.

[10] Genois A, Haig M, Roches AD, Sirard A, Le May S, McCuaig CC (2012). Case report of atopic dermatitis with refractory pruritus markedly improved with the novel use of clonidine and trimeprazine. *Pediatric Dermatology*.

[11] Weindruch R, Sohal RS (1997). Caloric intake and aging. *the New England Journal of Medicine*.

[12] Yagci G, Can MF, Ozturk E (2008). Effects of preoperative carbohydrate loading on glucose metabolism and gastric contents in patients undergoing moderate surgery: a randomized, controlled trial. *Nutrition*.

[13] Oh SG, Park YH (1976). Effect of fluid administration during the period of preoperative starvation on surgical patients. *Journal of the Korean Surgical Society*.

[14] Oh MS (2001). Ten golden rules of fluid therapy. *Korean Journal of Hepatology*.

[15] Kjeldsen-Kragh J, Haugen M, Borchgrevink CF (1991). Controlled trial of fasting and one-year vegetarian diet in rheumatoid arthritis. *The Lancet*.

[16] Iwashige K, Kouda K, Kouda M (2004). Calorie restricted diet and urinary pentosidine in patients with rheumatoid arthritis. *Journal of Physiological Anthropology and Applied Human Science*.

[17] Adeniyi FB, Young T (2012). Weight loss interventions for chronic asthma. *Cochrane Database of Systematic Reviews*.

[18] Ahmed T, Das SK, Golden JK, Saltzman E, Roberts SB, Meydani SN (2009). Calorie restriction enhances T-cell-mediated immune response in adult overweight men and women. *Journals of Gerontology A*.

[19] Kouda K, Tanaka T, Kouda M (2000). Low-energy diet in atopic dermatitis patients: clinical findings and DNA damage. *Journal of Physiological Anthropology and Applied Human Science*.

